# Where Does the Money Come From? Humanizing High Socioeconomic Status Groups Undermines Attitudes Toward Redistribution

**DOI:** 10.3389/fpsyg.2019.00771

**Published:** 2019-03-29

**Authors:** Mario Sainz, Rocío Martínez, Rosa Rodríguez-Bailón, Miguel Moya

**Affiliations:** ^1^Mind, Brain and Behavior Research Center, Department of Social Psychology, University of Granada, Granada, Spain; ^2^School of Psychology, University of Monterrey, Nuevo Léon, Mexico; ^3^Department of Social Psychology, Faculty of Psychology, University of Granada, Granada, Spain

**Keywords:** humanization, mechanization, high socioeconomic status groups, attributions of wealth, income redistribution

## Abstract

The concentration of wealth in the hands of a few at the expense of general impoverishment is a major problem in some modern societies. However, there is a general opposition to redistribution policies or to the application of a progressive taxation system. The goal of this research was to explore one factor that might drive the attitudes toward income redistribution: The (de)humanization of high socioeconomic status groups. Previous studies have shown that high socioeconomic status groups tend to be considered as unemotional machines without any concern for others. However, the consequences of mechanizing (vs. humanizing) high socioeconomic status on the interpretation of socioeconomic differences has not been explored yet. We considered that humanizing high socioeconomic status groups might have an unexpected negative effect on attitudes about income inequality and wealth concentration. Specifically, this research aims to determine how humanizing high socioeconomic status groups influences people’s perceptions of the group’s wealth and preferences for income redistribution. We conducted two studies in which we manipulated the humanity (mechanized vs. humanized in terms of their Human Nature traits) of a high socioeconomic status group. Results of these two studies showed that humanizing (vs. mechanizing) high socioeconomic status groups led to lower support for income redistribution/taxation of wealthy groups, through considering that the group’s wealth comes from internal sources (e.g., ambition) rather than external ones (e.g., corruption). These results were independent of the group’s likeability and perceived competence/warmth. The present research provides valuable insight about the possible dark side of humanizing high socioeconomic status groups as a process that could contribute to the maintenance of the status quo and the legitimation of income inequality in our societies.

## Introduction

The concentration of wealth in the hands of a few at the expense of general impoverishment is a major problem in some modern societies (Wilkinson and Pickett, 2010). Moreover, political corruption and wealthy individuals lobbying institutions for their self-interest are some of the major issues that concern citizens around the world (Transparency International, 2016). Despite these social issues’ importance, previous research has mainly focused on analyzing how groups at the bottom of society are perceived, while the perception of groups at the top of the socioeconomic hierarchy or people’s attitudes about wealth concentration of such groups has received much less attention (Bullock et al., 2008). In this regard, it is known that high socioeconomic status (SES) groups are sometimes dehumanized in a mechanistic way and therefore perceived as cold, superficial, and unemotional (Sainz et al., 2018). We believe consequences can arise based on possible humanization (vs. mechanization) of high-SES groups. Specifically, we believe that humanizing those who have more resources may help justify the *status quo* or reduce support for redistribution policies and that mechanizing high-SES groups might promote a negative perception of the sources of the group’s wealth, which could highlight class divisions in our societies. Due to these consequences’ social relevance, in the present research, we intended to experimentally analyze the influence of humanizing (vs. mechanizing) high-SES groups on the legitimation of the group’s wealth and people’s attitudes toward income redistribution.

In general, research has focused more on the perception of and attitudes toward low-SES groups. However, recent research has also explored the attitudes and perceptions of wealthy groups. For instance, a paper published by Horwitz and Dovidio (2017) analyzed the implicit and explicit attitudes of middle-SES participants toward high-SES groups. Results showed that middle-SES participants favored wealthy groups over middle-SES groups when implicit measures were used. However, participants did not openly favor wealthy groups when explicit means were used to assess attitudes about the group. In another set of studies, Van Doesum et al. (2017) compared prosociality toward low-, middle-, and high-SES groups. They found that high-SES groups elicited lower levels of prosociality compared to middle- or even low-SES groups. In other words, participants cared less about the needs or wishes of wealthy groups. Some studies have also compared how subtypes of wealthy groups (e.g., entrepreneurs, executives, people who inherit a lot of money) are perceived (e.g., Christopher et al., 2005; Sussman et al., 2014). This line of research suggests the existence of a possible link between the perception of a group and the sources of the group’s wealth. In this vein, the perceived source of the group’s wealth, such as inheritance or entrepreneurial success, influences the inference of traits of the target (e.g., entrepreneurs are perceived as being more open to experience or more competent than people who inherited their wealth; Christopher et al., 2005; Sussman et al., 2014).

Moreover, the SES of groups not only influences the ascription of some personality or competence traits, but also the humanized or dehumanized perception that people hold about a group (for a review, see Vaes et al., 2012; Haslam and Loughnan, 2014). Among the few studies that have analyzed the dehumanization of groups differing in SES, research conducted by Loughnan et al. (2014) showed that low-SES groups are considered as lacking human uniqueness (HU) traits (e.g., rationality, self-restraint) and are therefore dehumanized in an animalistic way. Moreover, another set of studies reported that high-SES groups are considered to have these HU traits but to lack human nature (HN) traits, such as emotionality or interpersonal warmth (Sainz et al., 2018). These results indicate that high-SES groups are considered as unemotional machines with a rigid behavior and without any ability to care about others. Indeed, we know that mechanizing (vs. humanizing) groups deeply influences the way people behave toward them. For instance, Bastian et al. (2010) demonstrated that groups perceived as having HN traits are considered to have more moral worth. By contrast, denying HN to a group leads to the perception that it is less deserving of moral treatment or less capable of rehabilitation after engaging in immoral behavior. This pattern of results highlights that humanity plays an important role in attributions about the behavior of a group and its consequences, with human (i.e., high HN) groups being punished to a lower extent than mechanized (i.e., low HN) groups after engaging in the same immoral behavior. Therefore, humanizing groups is likely to lead to a more permissive attitude toward outgroups by forgiving their undesirable behaviors through applying more relaxed moral standards. However, this affirmation is still under explored and more evidence is needed it in order to confirm changes in the attributional process underlying the (de)humanization of a group. Thus, we decided to explore this process in the context of socioeconomic comparisons with a group that has also being understudied in the literature about social class (i.e., high socioeconomic status groups): Specifically, we propose that humanizing (vs. mechanizing) high-SES groups may impacts on people’s perception of the group’s wealth and their attitudes toward its redistribution.

The perception that people have of socioeconomic groups could have an influence on their attitudes toward income inequality or the causal understanding of their socioeconomic differences. For instance, Tagler and Cozzarelli (2013) pointed out that a negative perception of poverty leads people to justify these groups’ belief that the situation is internally caused (e.g., “the poor are lazy”). Similarly, it can be expected that the (i.e., positive or negative) perception of high-SES groups could also shape the way the sources of the group’s wealth are perceived. Traditionally, lay theories about the causes of poverty or wealth typically differentiated between categories of factors (e.g., internal or external, controllable or incontrollable factors) used to explain the situation of the group (e.g., Cozzarelli et al., 2001; Bullock and Fernald, 2005; Weiner et al., 2010). Specifically, regarding the sources of wealth, Bullock and Fernald (2005) differentiated between internal causes (e.g., ambition or perseverance) and more external causes such as pull (e.g., corruption, lobbying institutions), luck (e.g., winning the lottery) or inheriting from relatives, among others: More external attributions of wealth tend to lead people to consider the wealth of the groups as unfairly acquired; while considering that the wealth is the product of internal rather than external causes, is likely to lead to a perception that the situation of wealthy people and groups is fair and legitimate. Additionally, the type of attributions that people make about the wealth of a group have a great impact on the attitudes they hold about social policies (e.g., Alesina and La Ferrara, 2005; Bullock and Fernald, 2005). For example, thinking about wealth as having an internal cause leads people to show less support for income redistribution or taxation; by contrast, considering that the wealth results from corruption or from being born in a wealthy family (i.e., an external cause) leads to a more positive attitude toward progressive taxation or other policies to redistribute wealth.

Overall, based on these previous evidences, we expected the attribution of humanity (humanizing vs. mechanizing) to high-SES groups to influeence how legitimate or illegitimate people could perceive the process of becoming wealthy and also people’s attitudes toward wealth redistribution. Specifically, we predicted that people’s attributions about the causes of wealth would be influenced by their perception of wealthy groups. For instance, Tagler and Cozzarelli (2013) showed that a negative perception of poverty is associated with a higher endorsement of internal attributions of poverty. Similarly, but regarding rich people, we proposed that humanizing high-SES groups would lead people to consider that they acquired their wealth by internal means (e.g., effort, perseverance) rather than from external sources (e.g., corruption, dishonesty). This process of attributing wealth to internal causes (e.g., hard work, ambition) implies that the wealth of the groups is fair and deserved. By contrast, we expected the mechanization of high-SES groups leads to attributing their wealth to external rather than internal means. Specifically, we expected people to consider that machine-like groups do not care about others and lack a sense of morality, which is likely to make them more willing to use any kind of strategy to reach a wealthier position. This ultimately implies that the position of the group is unfair and less deserved.

Furthermore, Bullock and Fernald (2005) also reported that a positive perception of wealthy groups led to a lower demand for taxation of high-SES groups. Thus, it can also be inferred that a human perception of wealthy groups decreases supports for economic redistribution and progressive taxation. This is consistent with the idea that humanized groups (i.e., those with high HN) are considered as having a higher standard of moral responsibility (Bastian et al., 2010). Such groups are considered as being unable to engage in immoral behaviors (e.g., corrupt practices or lobbying institutions for their self-interest); by contrast, mechanized groups are seen as likely not to restrain from engaging in these types of illegitimate behaviors that led them to their wealthy position. Along the same lines, we hypothesized that humanizing (vs. mechanizing) high-SES groups would lead to a legitimate perception of wealth (e.g., internally caused and fairly perceived) and consequently to a lower demand for income redistribution. In short, we proposed that, in the context of our study, the humanization of wealthy groups would have a paradoxical negative effect by promoting perceptions and attitudes that contribute to favor a more unequal society. This possible negative consequence that have being understudied before, was tested on two experimental studies in which we analyze how humanization can encourage the maintenance of unequal distribution.

## Study 1

The main goal of this study was to test whether humanizing (vs. mechanizing) high-SES groups affects the source of the group’s wealth, and people’s attitudes toward redistribution (i.e., redistribution preferences and progressive taxation). Specifically, we expected the wealth of high-SES groups that were humanized (i.e., high in HN) to be attributed to internal rather than external causes, than that of high-SES groups seen as mechanized (i.e., low in HN; Hypothesis 1). Regarding attitudes toward income redistribution, we expected the fact of humanizing high-SES groups to lead to a lower support for both income redistribution (Hypothesis 2a) and taxation of the wealthiest groups (Hypothesis 2b) compared to the mechanization of high-SES groups. Additionally, we expected wealth legitimation (index of internal and external attributions) to mediate the relationship between (de)humanization of the group and attitudes toward economic redistribution (Hypothesis 3). All the materials used in the studies and the corresponding data can be found online (osf.io/es84x).

### Participants and Procedure

Participants were students who attended university libraries in a city in southern Spain. They were asked to participate in a study about the perception of groups. The study received approval from the ethics committee of the University of Granada and consent was obtained from the participants before providing the questionnaire. Sample sized was calculated using G-power analysis (Faul et al., 2009) for an independent *t-test* (two tails, α = 0.05, 80% Power, medium-small effect size *d* = 0.40, required minimum *n* = 200). The final sample was composed of 274 participants (140 women, 129 men, *M_age_* = 23.94, *SD* = 4.84). Once participants agreed to participate voluntarily in the study, they were presented with a questionnaire that included the following sections:

#### Manipulation of High-SES Humanity

In order to manipulate the humanity of a high-SES target, participants were presented with a fictitious newspaper article about a scientific article published in a well-known journal of social psychology. Participants read that the authors of the article had analyzed the traits associated to different groups of society. Next, they were told that the aim of our research was to analyze how people perceived the group that appeared in the article. Participants were assigned randomly to one of the two conditions: In both conditions, they read a short description of a group considered as high SES. We provided a few details about the group members’ high income, education and status. After reading this information, the description of the group varied regarding the ascription of HN traits in order to manipulate its humanity (i.e., mechanized vs. humanized; see materials from Martínez et al., 2015). In the mechanized condition, the group was described as being machine-like/lacking HN (e.g., with a passive and superficial attitude regarding things that happen around it, and with a rigid and cold behavior); in the humanized condition the group was described as being human/having HN traits (e.g., an active and reflexive attitude and a flexible and warm behavior). After reading the description of the group, participants answered some manipulation check questions on the SES of the group (“What is the SES of the group described in the text?”; single Likert item from 1 – Poor – to 10 – Rich) and a manipulation check on HN level (e.g., “To what extent is the group “emotional, flexible and open-minded”?”; α = 0.96, two Likert items from 1 – Not at all – to 7 – Completely). Additionally, we measured the HU attributed to the group (e.g., “To what extent is the group “rational, civic-minded and educated”?”; α = 0.75, two Likert items from 1 – Not at all – to 7 – Completely) to control for this dimension of humanity.

#### Legitimation of the Wealth of High-SES Groups

We included a scale for the source of the group’s wealth (Bullock and Fernald, 2005). The scale included 22 items that differentiated between four dimensions or causes of wealth: Perseverance/ambition (e.g., ability, hard work; 8 items, α = 0.81); corruption/pull (e.g., ruthlessness, networking; 6 items, α = 0.72); fatalism/luck (e.g., winning the lottery; 4 items, α = 0.41); and privilege/inheritance (e.g., attending elite universities; 4 items, α = 0.67). As in the original paper (Bullock and Fernald, 2005), the last two factors were less consistent and showed lower reliability. Therefore, we decided to run a factor analysis to simplify the structure of the scale. Results showed that a first factor explained 22.49% of the variance and included mainly items related to internal causes (e.g., ambition; 9 items, α = 0.82); the second factor explained 15.71% of the variance and included items related to external causes (e.g., having the right contacts; 13 items, α = 0.77). This two-factor structure allowed us to analyze how legitimate the wealth of the groups was perceived to be by comparing the amount of internal vs. external attributions that participants made. We computed an index of the legitimation of wealth source by subtracting internal from external causes; lower scores indicated that the wealth of the group was perceived as having been acquired by external means.

#### Attitudes Toward Redistribution

To analyze to what extent people were prone to support the redistribution of the wealth of this group, we included two items (e.g., “The government should redistribute wealth through heavily taxing this group”; from 1 – Totally disagree – to 7 – Completely agree, α = 0.86) adapted from Dawtry et al. (2015). Additionally, we included a single item on taxation adapted from Gross et al. (2017). Participants were asked what percentage of taxes the group should pay from 0 (i.e., no taxes) to 100 (i.e., the full amount of the group’s income per month). Given the high correlations between these last two measures, we computed an index of support for redistribution (higher scores indicated greater support for redistribution) by averaging the scores on these two items for the mediation analysis (*r* = 0.50, *p* ≤ 0.001). Finally, participants reported some demographic information (e.g., age, gender) and were thanked for participating in the study and debriefed.

### Results

First, we analyzed the results of the manipulation check questions. Regarding the SES of the group, we calculated a one-sample *t-test* to verify that participants ascribed high SES to the groups described in both conditions. Result indicated that the groups were perceived as having high SES (*M* = 8.29, *SD* = 1.41, significantly above the mean of the scale, *t*_(273)_ = 15.08, *p* ≤ 0.001). Additionally, participants assigned to the mechanized condition reported that the group described had lower HN levels (*M* = 1.90, *SD* = 0.98) than did participants assigned to the humanized condition (*M* = 5.39, *SD* = 1.34, *t*_(272)_ = -24.3, *p* ≤ 0.001, 95% CI [-3.77, -3.21], Hedges’ g_s_ = 2.95), confirming the effectiveness of the manipulation.

Second, we computed simple differences regarding wealth legitimation and support for redistribution/taxation that people hold as a function of the condition (see Table 1). Results seem to indicate that the mechanized group’s wealth was perceived as more illegitimate (index of wealth legitimation) than that of the humanized group (Hypothesis 1). Specifically, results indicated that more internal attributions were made in the case of the humanized (vs. mechanized) group, and more external attributions were made for the mechanized (vs. humanized) group. In relation with the general index of support for redistribution, results indicated that people were less willing to redistribute income from the humanized group than from the mechanized group. Specially, results indicated that people were more willing to redistribute income by asking for higher taxation of the rich (Hypothesis 2b) than by supporting income redistribution *per se* (Hypothesis 2a).

**Table 1:** Differences between conditions (mechanized vs. humanized group) in legitimation of wealth and the support for redistribution (i.e., support for income redistribution and support for higher taxation of high-SES groups) variables included in studies 1 and 2.

	Mechanized *Mean (SD)*	Humanized *Mean (SD)*	*t*	*p*	*95% CI*	*Hedges’ g_s_*
**Index of wealth legitimation**						
Study 1	-0.70 (*1.36*)	0.09 (*1.32*)	*t*_(272)_ = 4.91	0.00	[0.48, 1.12]	0.59
Study 2	-0.36 (*1.29*)	0.29 (*1.25*)	*t*_(337)_ = 4.70	0.00	[0.38, 0.92]	0.51
**Internal attributions**						
Study 1	4.02 (*1.03*)	4.47 (0*.90*)	*t*_(272)_ = -3.82	0.00	[-0.68, -0.22]	0.46
Study 2	4.18 (0*.92*)	4.52 (0*.74*)	*t*_(337)_ =-3.77	0.00	[-0.52, -0.12]	0.41
**External attributions**						
Study 1	4.72 (0*.79*)	4.37 (0*.92*)	*t*_(272)_ = 3.37	0.00	[0.14, 0.15]	0.41
Study 2	4.54 (0*.81*)	4.24 (0*.93*)	*t*_(337)_ = 3.24	0.00	[0.15, 0.49]	0.34
**Index of support for redistribution**						
Study 1	0.13 (0*.80*)	-0.11 (0*.92*)	*t*_(271)_ = -2.29	0.02	[-0.03, -0.45]	0.27
Study 2	0.10 (0*.87*)	-0.12 (0*.88*)	*t*_(337)_ = -2.32	0.02	[-0.03, -0.41]	0.25
**Support for redistribution**					
Study 1	5.07 (*1.62*)	4.70 (*1.73*)	*t*_(271)_ = 1.82	0.07	[-0.03, 0.77]	0.22
Study 2	5.14 (*1.37*)	4.85 (*1.35*)	*t*_(337)_ = 1.94	0.05	[-0.00, 0.58]	0.21
**Support for higher taxation**					
Study 1	50.53 (*13.99*)	46.53 (*16.37*)	*t*_(261)_ = 2.13	0.03	[0.30, 7.71]	0.26
Study 2 (index)	18.94 (*15.52*)	15.20 (*16.40*)	*t*_(297)_ = 2.02	0.04	[0.10, 7.37]	0.23


Finally, we conducted mediation analyses with humanity (machine = 0, human = 1) as the predictor of the index of support for redistribution through wealth legitimation using the PROCESS macro (bootstrapping 10,000 interactions, 95% confident intervals) by Hayes (2013). Results indicated that wealth legitimation was a significant mediator of the relationship between humanity and preferences for redistribution (see Table 2). This indirect effect remained significant while performing the same analysis with separate measures and even after controlling for HU (Appendix S1, S2). In short, we found empirical evidence that humanizing high-SES groups leads to the legitimation of the group’s wealth, which in turn decreases people’s support for redistributing wealth, in line with our exploratory Hypothesis 3.

**Table 2:** Total, direct, and indirect effects with standard error (*SE*) of the mediation of wealth legitimation (index) on the relationship between (de)humanization and the support for redistribution (index) for studies 1 and 2.

	VD: Index of general support for redistribution						
	Effect (*SE*)	*95% CI*	*p*		Effect (*SE*)	*95% CI*	*p*
**Total effect**							
Study 1	-0.24 *(0.10)*	[-0.45, -0.03]	0.02	Study 2	-0.22 *(0.09)*	[-0.41, -0.03]	0.02
**Direct effect of dehumanization**							
Study 1	0.02 *(0.09)*	[-0.17, 0.20]	0.90	Study 2	0.03 *(0.09)*	[-0.21, 0.14]	0.65
**Indirect effect of wealth legitimation**							
Study 1	-0.25 *(0.06)*	[-0.37, -0.14]	<0.001	Study 2	-0.18 *(0.04)*	[-0.27, -0.10]	<0.001


### Discussion

In this study we analyzed how the humanization of high-SES groups, compared to their mechanistic dehumanization, influences the perception of the sources of their wealth and consequently people’s attitudes toward wealth redistribution. Results indicated that the wealth of a group described as human (e.g., warm and open-minded) vs. mechanized (e.g., cold and inflexible) was considered as more legitimate, as it was supposed to originate from internal sources (e.g., effort, ambition) instead of external ones (e.g., inheritance, corruption); this led to a lower support for redistributing income policies (general index). Even when results indicated that differences applied on the support for higher taxes, no differences were found on the redistribution policies. It might be possible that people were willing uniquely to punish the group (i.e., more taxes) but not to redistribute income among others groups. However, this result needs of replication before interpreting possible differences among measures of redistribution.

In general, results indicated that dehumanizing high-SES groups seems to promote the motivation for redistribution as a consequence of the perception that cold and rigid groups acquire their wealth from external and unfair sources; by contrast, humanizing high-SES groups seems to show the opposite effect. These results suggest that in the context of hierarchical upward comparisons, humanization can have a dark side by legitimating an unequal situation, and dehumanization promotes the group’s punishment. The implications that could arise from these findings on interclass relations might be severe. Therefore, we decided to replicate the study to have confirmatory evidence. Moreover, while replicating the study, we wanted to improve the manipulation of humanity that we used (Martínez et al., 2015), and we wanted to rule out alternative factors, such as the competence/warmth ascribed to high-SES groups (Durante et al., 2017). It might be possible that the description of the humanized group may have given a better or more competent impression of it than that of the mechanized group. Considering this, we tried to overcome these potential problems in Study 2, by improving the manipulation and controlling for these possible confounders in order to replicate Study 1.

## Study 2

We designed a second study to replicate the findings of Study 1. The main change made in Study 2 was to include in our manipulation only human traits that differed in the level of HN, controlling for valence and HU. We also included a measure of competence/warmth of high-SES groups to control by these possible confounders. Finally, we used a general population sample instead of a college sample.

Our hypothesis was pre-registered and can be found online (osf.io/m2pqy). We expected to find differences between conditions (i.e., mechanized vs. humanized), with a more legitimate perception of the group wealth for the humanized group compared to the mechanized group (Hypothesis 1). Regarding attitudes toward income redistribution, we expected to find a lower support for income redistribution regarding the humanized group than regarding the mechanized group (Hypothesis 2). Finally, we expected wealth legitimation to mediate the relationship between (de)humanization and attitudes toward redistribution policies (Hypothesis 3).

### Pilot Study

We ran a pilot study to improve the descriptions of the mechanized and the humanized groups. Our main goal was to select personality traits that allowed us to create descriptions that only differed in ascribed HN (low or high HN) but not in valence or HU. We recruited 38 participants (26 females, 12 males, *M_age_* = 23.24, *SD* = 5.39) at a bus station in a city of southern Spain. Once participants agreed to participate in a study about word comprehension, they were asked to rate 80 personality traits following the same procedure as that proposed by Ferrari et al. (2016). Specifically, participants were asked to indicate to what extent each trait was representative of HN (“To what extent does the following word represent a human nature trait and is not applicable to robots or machines?”), HU (“To what extent does the following word represent a uniquely human trait, which is therefore not present in other animal species?”), and the valence of the traits (“To what extent is the following word positive or negative when applied to a group of people?”). Answers were provided on a 5-point Likert scale with higher scores indicating the words were more representative of HN and HU and more positively evaluated. Finally, we selected 10 high (*M* = 3.61, *SD* = 1.04) and 10 low (*M* = 2.73, *SD* = 0.73) HN traits that differed significantly, *t*_(37)_ = 3.72, *p* ≤ 0.001, 95% CI [0.40, 1.35], Hedges’ g_av_ = 0.96. No differences were found regarding the valence of the high-HN traits (*M* = 2.90, *SD* = 0.33) when compared to the low-HN traits (*M* = 2.91, *SD* = 0.36), *t*_(37)_ = -0.231, *p* = 0.819, 95% CI [-0.14, 0.11] or regarding the ascribed level of HU (*M* = 3.19, *SD* = 0.71; *M* = 3.08, *SD* = 0.72 for high and low HU, respectively), *t*_(37)_ = -1.06, *p* = 0.295, 95% CI [-0.10, 0.33]. This selection of traits allowed us to build a fictitious description of a high-SES group that differed only in the ascribed level of HN (high vs. low) while controlling for the valence and ascribed level of HU (see Table 3).

**Table 3:** Original (in brackets) and translated version of the traits selected in the pilot study for the manipulation of high-SES humanity (low and high-HN traits, both positive and negative) in Study 2.

	Low HN (Machine-like)	High HN (Human-like)
Positive traits	Analytic *(Analítico/a)*	Open-minded *(Abierto/a de mente)*
	Competent *(Competente)*	Emotional *(Emocional)*
	Methodical *(Metódico/a)*	Receptive *(Receptivo/a)*
	Organized *(Organizado/a)*	Sensitive *(Sensible)*
	Precise *(Preciso/a)*	Passionate *(Pasional)*
Negative traits	Cold *(Frío)*	Jealous *(Celoso/a)*
	Unemotional *(Poco emocional)*	Nervous *(Nervioso/a)*
	Inflexible *(Inflexible)*	Impatient *(Impaciente/a)*
	Insensitive *(Insensible)*	Envious *(Envidioso/a)*
	Strict *(Estricto/a)*	Indiscreet *(Indiscreto/a)*


### Participants and Procedure of the Main Study

Participants were selected among people who were at the bus station of a city in southern Spain. Sample size was calculated for an independent *t-test* (α = 0.05, 80% Power, *d* = 0.40, minimum *n* = 200). The final sample was composed of 339 participants (239 women, 100 men, *M_age_* = 25.54, *SD* = 9.47). The study was approved by the ethics committee of the University of Granada and consent was obtained from the participants. Once participants had agreed to participate, they were presented with a paper and pencil questionnaire that contained the following measures in this order:

#### Manipulation of the Humanity of High-SES Groups

Following the same procedure as in Study 1, participants read a description of a humanized vs. mechanized fictitious high-SES group using the personality traits selected in the pilot study. After reading the description of the group, participants answered a manipulation check question about the group’s SES (“What is the SES of the group described in the text?”; from 1 – Poor – to 3 – Rich), and its perceived HN level (e.g., “To what extent is the group “emotional, flexible and open-minded”?”; from 1 –Not at all – to 7 – Completely, two items, α = 0.76). Additionally, we included one item that measured the perceived competence of the group (e.g., “To what extent is the group “competent, skillful and intelligent”?”) and one item that measured its warmth (e.g., “To what extent is the group “warm, affectionate and tender”?”). Answers were provided on a Likert-type scale from 1 – Not at all – to 7 – Completely.

#### Legitimation of High-SES Group Wealth and Attitudes Toward Wealth Redistribution

We included the same measures about perceived wealth source as in Study 1. Regarding attitudes about redistribution, we slightly modified the previous measures of redistribution by including three items instead of two (from 1 – Totally disagree – to 7 – Completely agree, α = 0.72) in order to include an additional reverse item. Additionally, participants were asked to estimate the amount of taxes the group should pay and the taxes the group was currently paying using a percentage, from 0% (no taxes at all) to 100% (all their monthly income). These two questions allowed us to create an index of increasing/decreasing taxes (i.e., taxes respondents estimated what the group should pay compared to the taxes that it was currently paying). As in the previous study, an index of general support for income redistribution was created (*r* = 0.41, *p* ≤ 0.001). Finally, participants provided some demographic information (e.g., age, gender) and were thanked for participating in the study and debriefed.

### Results

First, we analyzed the results of the manipulation check questions. Groups in both conditions were perceived as having high-SES (*M* = 2.94, *SD* = 0.26, significantly above the mean point of the answer scale, *t*_(338)_ = 66.68, *p* ≤ 0.001). Additionally, we found the expected differences in the attribution of HN to the humanized group (*M* = 4.80, *SD* = 1.35) and the mechanized group (*M* = 2.38, *SD* = 1.01, *t*_(337)_ = 81.65, *p* ≤ 0.001, 95% CI [2.17, 2.68], Hedges’ g_s_ = 2.02), which confirmed the effectiveness of our manipulation.

Second, we computed the differences between both experimental conditions for the measures separately (see Table 1). Simple *t-test* comparisons indicated that participants considered the mechanized group’s wealth more illegitimate, and they were more willing to redistribute it than the humanized group’s wealth. These results replicated previous findings, supporting hypotheses 1 and 2. In short, the humanization of wealthy groups contributes not only to legitimating their wealth, but also to decreasing the perception that their wealth should be redistributed.

Finally, we conducted mediation analyses with humanity (machine = 0, human = 1) as the predictor of the support for redistribution through the mediational effect of wealth legitimation as in Study 1, using the PROCESS macro (bootstrapping 10.000 interactions, 95% confident intervals) by Hayes (2013). Results indicated that wealth legitimation was a significant mediator of the relationship between humanity and the support for redistribution (see Table 2), supporting our Hypothesis 3. This indirect effect remained significant while performing the same analysis with separate measures and after controlling for competence and warmth (Appendix S1, S3).

### Discussion

The results of this study provided confirmatory evidence of how humanization vs. mechanization of high-SES groups affects people’s perceived source of wealth and their support for economic policies related to redistribution. Humanizing (vs. mechanizing) high-SES groups led people to consider that the wealth of the groups resulted from their hard work and their personal ambition rather than from their corrupt practices or an inheritance. Lastly, this led participants to justify income inequality. Participants supported in a lower extent income redistribution and considered that humanized (vs. mechanized) high-SES groups fulfill their obligations when paying taxes. This pattern of results was found even when the descriptions of the humanized and mechanized condition were matched in valence, and also when controlling for competence and warmth, supporting our hypothesis about the importance of humanizing high-SES groups for the justification of inequality, above and beyond other social dimensions of comparison.

## General Discussion

In the present research we analyzed the consequences of humanizing (vs. mechanizing) high-SES groups on the perception of the group’s wealth and on the attitudes people hold about income redistribution policies. Results indicated that humanizing high-SES groups leads to more internal attributions of the source of the group’s wealth and hence lower support for redistributing the group’s wealth. Mechanizing high-SES groups leads to more external attributions of the source of the group’s wealth and hence a willingness to redistribute this group’s wealth. In short, in the context of our study, humanizing people with an advantaged position helped justify the unequal *status quo*, and mechanizing them seems to promote redistribution. The results in both cases seem to be due to the perceived attributions of the group’s wealth.

Previous studies have analyzed the perception of different types of wealthy groups (e.g., Christopher et al., 2005; Sussman et al., 2014), high-status groups (e.g., Capozza et al., 2011) or high-status professions (Iatridis, 2013). Moreover, the role of hierarchy-based variables, such as meritocracy (García-Sánchez et al., 2018) or anti-egalitarian attitudes (Kteily et al., 2017), on the perception of and attitudes toward income inequality have also been discussed. Yet, as far as we know, the role and consequences of (de)humanizing high-SES groups on the justification of inequalities has not been studied before: These results highlight that having a humanized perception of groups with an advantaged position can have negative consequences regarding the maintenance of income inequality. By contrast, mechanizing high-SES groups seems to have the opposite effect, by favoring income redistribution as a consequence of perceiving the wealth of the groups as illegitimate. This possible dark side of humanization has been identified before, for example in the medical context, where humanizing patients made it more difficult for professionals to cope with the suffering of their patients (Haque and Waytz, 2012), but not in the context of hierarchical differences between groups.

Furthermore, the higher taxation of the mechanized (vs. humanized) group could be explained based on the previous evidence. For example, Bastian et al. (2010) showed that groups considered as lacking HN traits (i.e., machine-like) are perceived as having less moral values or acting in a less prosocial way, which leads people to be more prone to punish them compared to groups with HN traits. In this context, redistribution policies, especially taxation, may be understood as a way to punish groups that have a privileged position rather than a legitimate means to reduce income inequality. Therefore, people are likely to demand a stricter financial pressure for machine-like high-SES groups because they are considered to break the social norms, for example using corrupt practices (i.e., external attributions) for their own benefit.

Although mechanizing a group seems to have negative consequences for groups that are dehumanized (e.g., Bastian et al., 2010), our results also showed that humanization can have some detrimental consequences for the well-being of the entire society. One of the possible consequences that arise from the two studies presented here is that humanizing high-SES members may act as a blindfold that undermines our tendency to act in favor of a more equal society. A humanized perception of wealthy groups may lead people to minimize the importance of standing up against corrupt practices or political influence (i.e., external attributions) committed by wealthy groups, for example. It may also contribute to the denial of the use of non-ethical strategies (e.g., money laundering, use of tax havens) by the rich to avoid paying taxes and help build a better society. As Bastian et al. (2010) pointed out, humanized groups are positively regarded because of their ascribed morality. Therefore, it might be possible that by humanizing wealthy groups we are likely to assume that they have a high moral standard and divert our attention from the potential unethical behaviors they perform. These potential social implications of humanizing privileged groups, which could be addressed in futures studies, would definitely contribute valuable information about the mechanisms that might help maintain the *status quo.*

Undeniably, this research has some limitations. First, we focused only on the perception of external vs. internal sources of wealth without considering other categorizations that include, for instance, the ability of groups to control their sources of wealth (e.g., Weiner et al., 2010; Testé, 2017). By including the control dimension, future studies will be able to compare the extent to which humanized vs. mechanized groups are considered to have reached their wealthy position through internal and controllable means (e.g., personal effort) or external and incontrollable means (e.g., winning the lottery), and finally how this affects attitudes about redistribution. In addition, we only assessed the consequences of (de)humanizing wealthy groups by providing participants with information about the SES of the groups. Future studies could provide more information about what specific traits on the HN dimension are currently driving the present results. This will contribute to disentangle if redistribution attitudes are driven more by the lack of prosociality of the mechanized groups or by other factors such as the lack of emotionality of the machine-like group. Moreover, attitudes toward redistribution may be modulated not only by the humanity of the groups but also by the source of their resources. As found by previous studies (Christopher et al., 2005), differences may arise between wealthy groups such as entrepreneurs, people who inherited their wealth, or people who won the lottery. Undoubtedly, comparing the humanity ascribed to subtypes of wealthy groups and the respective attitudes about the redistribution of their wealth will provide valuable insight to the study of perceived wealth inequality and system justification.

Future studies could also explore how the present pattern of results is modulated by the subjective perception of the gap between the rich and the poor. Previous studies have found that people show less support for income redistribution when the perceived level of inequality is high (Heiserman and Simpson, 2017). Hence, our pattern of results may be modulated by the perceived level of inequality. Furthermore, it would be also interesting to explore the individual factors that lead participants to hold a human perception of wealthy groups. Beliefs about social mobility or the effects of hierarchy-based ideologies, such as social dominance orientation or anti-egalitarian attitudes (e.g., Kteily et al., 2017; Rodríguez-Bailón et al., 2017) may promote this human perception of wealthy groups. Finally, even when we have focused on the role of humanizing high-SES groups in the socioeconomic domain, positive consequences could be also explored when one humanizes these groups. Specifically, the positive consequences might be revealed if the previously identified dehumanization of low and high-SES is overcome (Loughnan et al., 2014; Sainz et al., 2018). The humanization of the extremes of the socioeconomic ladder might render a more cohesive society, which could help counter the downward spiral of classist attitudes that undermine social cohesion and act against possible collective actions for the benefit of all. Future studies should address these issues due to their social relevance.

To sum up, in the current context where income inequality is increasing in some countries, more attention should be devoted to analize how wealthy individuals and groups are perceived in terms of their humanity. Wealth may be something that we appreciate and desire, but it can also be the trigger that promotes evil or greedy behaviors that contribute to exploitation of others. Our results add to the previous literature on the importance of humanization on how wealth and wealthy groups are understood and perceived by highlighting the possible consequences of humanizing advantaged groups.

## Data Availability

All datasets generated for this study are included in the manuscript and/or the Supplementary Files.

## Ethics Statement

This study was carried out in accordance with the guidelines stated by the Vice-rectory of Research and Scientific Policy of the University of Granada, fulfilling the requirements of informed consent and data protection stated by the Spanish Organic Law 15/1999. The protocol was approved by the Ethics Committee for Research of the University of Granada (No. 170/CEIH/2016).

## Author Contributions

MS conceived the presented idea, collected the data and analyzed the results. All authors verified the analytical methods, discussed the results and contributed to the final manuscript.

## Conflict of Interest Statement

The authors declare that the research was conducted in the absence of any commercial or financial relationships that could be construed as a potential conflict of interest.
