# Supplementary material for: Where Does the Money Come From? Humanizing High Socioeconomic Status Groups Undermines Attitudes Toward Redistribution
Source: Front Psychol. 2019 Mar 29;10:771. doi: 10.3389/fpsyg.2019.00771 (PMC6450225; doi:10.3389/fpsyg.2019.00771)
Supplement: Supplementary file 1 [file Data_Sheet_1.PDF]

Supplementary Material

Appendix S1

We computed the same mediation analysis by using the separate variables included in studies 1 and 2. In each study we conducted two mediation analyses with humanity (machine = 0, human = 1) as the predictor of the support for redistribution (Dependent 1) and taxation (Dependent 2) through the index of wealth legitimation using the PROCESS macro (bootstrapping 10,000 interactions, 95% confidence intervals) by Hayes (2013). Results indicated that the index of wealth legitimation was a significant mediator on the relationship between humanity and preferences for redistribution in the analyses (see Table 4). In short, we found empirical evidence about how humanizing high-SES groups predicts a more legitimate perception of the group’s wealth, which leads to a lower support for redistributing wealth or increasing the taxes that the group should pay, supporting our exploratory Hypothesis 3.

Table 4. Total, direct, and indirect effect with standard error (*SE*) for the mediation of the index of wealth legitimation in the relationship between (de)humanization and support for income redistribution (Dependent 1) and support for higher taxation (Dependent 2), for studies 1 and 2.

	VD$_1$: Support for Redistribution			VD$_2$: Support for Higher Taxation		
	IE (*SE*)	*95% CI*	*p*	IE (*SE*)	*95% CI*	*p*
Total Effect						
Study 1	-.37 *(.20)*	[-.77, .03]	.07	-4.00 *(1.88)*	[-7.71, -.30]	.03
Study 2	-.29 *(.14)*	[-.57, .01]	.05	-3.74 *(1.85)*	[-7.37, -.10]	.04
Direct Effect						
Study 1	.10 *(.19)*	[-.27, .46]	.59	-.59 *(1.80)*	[-4.14, 2.97]	.74
Study 2	-.04 *(.14)*	[-.32, .24]	.77	-1.01 *(1.80)*	[-4.55, 2.53]	.57
Indirect Effect						
Study 1	-.47 *(.11)*	[-.70, -.26]		-3.42 *(.84)*	[-5.19, -1.88]	
Study 2	-.25 (.06)	[-.38, -.13]		-2.73 *(.80)*	[-4.51, -1.35]	

Appendix S2

Due to the differences between the humanized group (*M* = 5.20, *SD* = 1.03) and the mechanized group (*M* = 4.35, *SD* = 1.42, $t_{(272)}$ = 5.63, $p \leq .001$, 95% CI [.55, 1.14], Hedges' g_s = .68) in ascribed HU, we decided to perform an alternative mediation analysis controlling for the ascribed level of HU between both conditions. Results indicated that, after controlling for HU, the indirect effect remained significant (see Table 5).

Table 5. Total, direct, and indirect effect with standard error (*SE*) for the mediation of the index of wealth legitimation in the relationship between (de)humanization, support for income redistribution (Dependent 1) and support for higher taxation (Dependent 2), controlling for the ascribed HU to the high-SES group in Study 1.

	VD_1: Support for Redistribution			VD_2: Support for Higher Taxation		
	IE *(SE)*	*95% CI*	*p*	IE *(SE)*	*95% CI*	*p*
Total Effect	-.24 *(.21)*	[-.66, .18]	.26	-2.56 *(1.96)*	[-6.43, 1.30]	.19
Direct Effect	.08 *(.19)*	[-.30, .46]	.69	-.35 *(1.87)*	[-4.03, 3.32]	.85
Indirect Effect	-.32 *(.11)*	[-.54, -.12]		-2.21 *(.73)*	[-3.70, -.82]	

Appendix S3

To isolate the effect of the attribution of HN (low HN/machine and high HN/human) with respect to the well-known dimensions of the stereotype content model (i.e. competence and warmth), we decided to perform an alternative mediation analysis controlling for the competence and warmth ascribed to high-SES groups (see Table 6). Results revealed that, although the effect was reduced, the indirect effect remained significant even when controlling for the ascribed competence and warmth of the groups described as having high SES.

Table 6. Total, direct, and indirect effect with standard error (*SE*) of the mediation of the index of wealth source in the relationship between (de)humanization and support for income redistribution (Dependent 1) and support for higher taxation (Dependent 2), controlling for the competence and warmth of high-SES groups in Study 2.

	VD_1: Support for Redistribution			VD_2: Index of Taxation		
	IE (SE)	$^{95\%}$ *CI*	*p*	IE (SE)	$^{95\%}$ *CI*	*p*
Total Effect	-.15 (.16)	[-.47, .16]	.34	-3.80 (2.01)	[-7.76, .16]	.06
Direct Effect	.03 (.15)	[-.33, .27]	.85	-2.11 (1.91)	[-5.85, 1.64]	.26
Indirect Effect	-.12 (.06)	[-.24, -.02]		-1.69 (.81)	[-3.47, -.27]	
